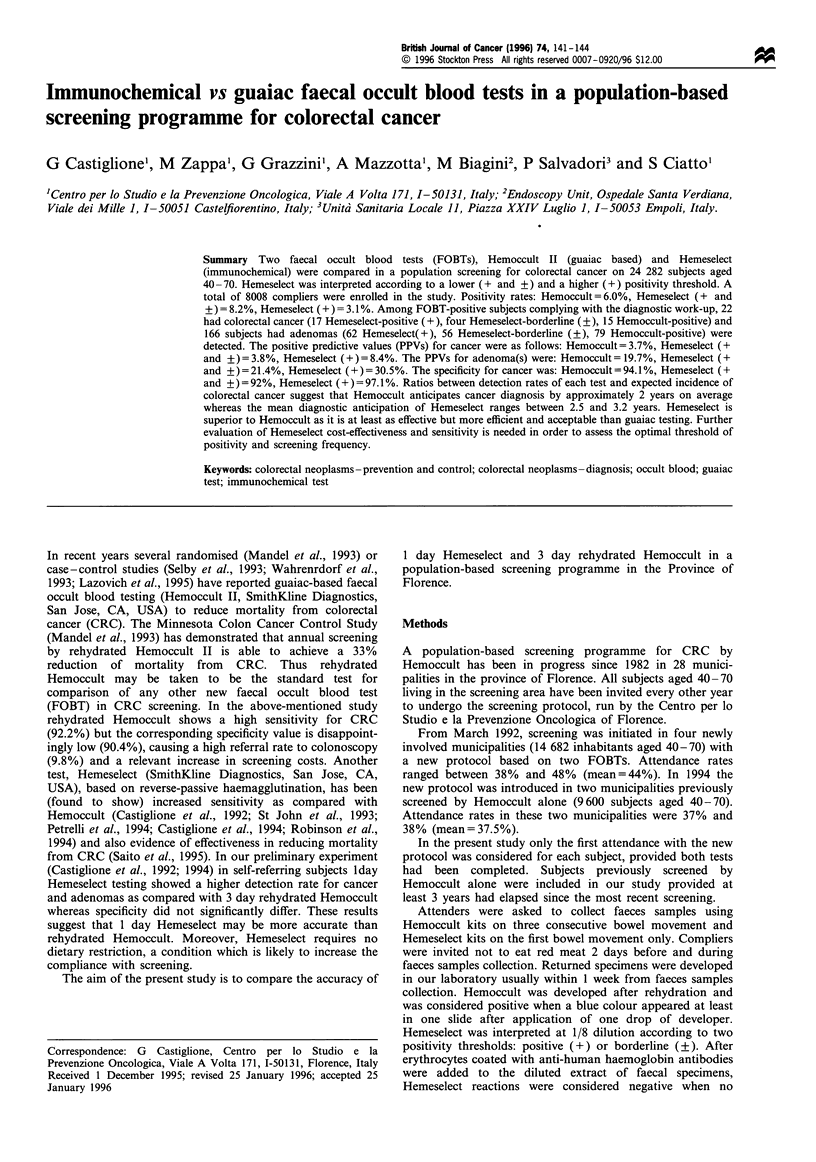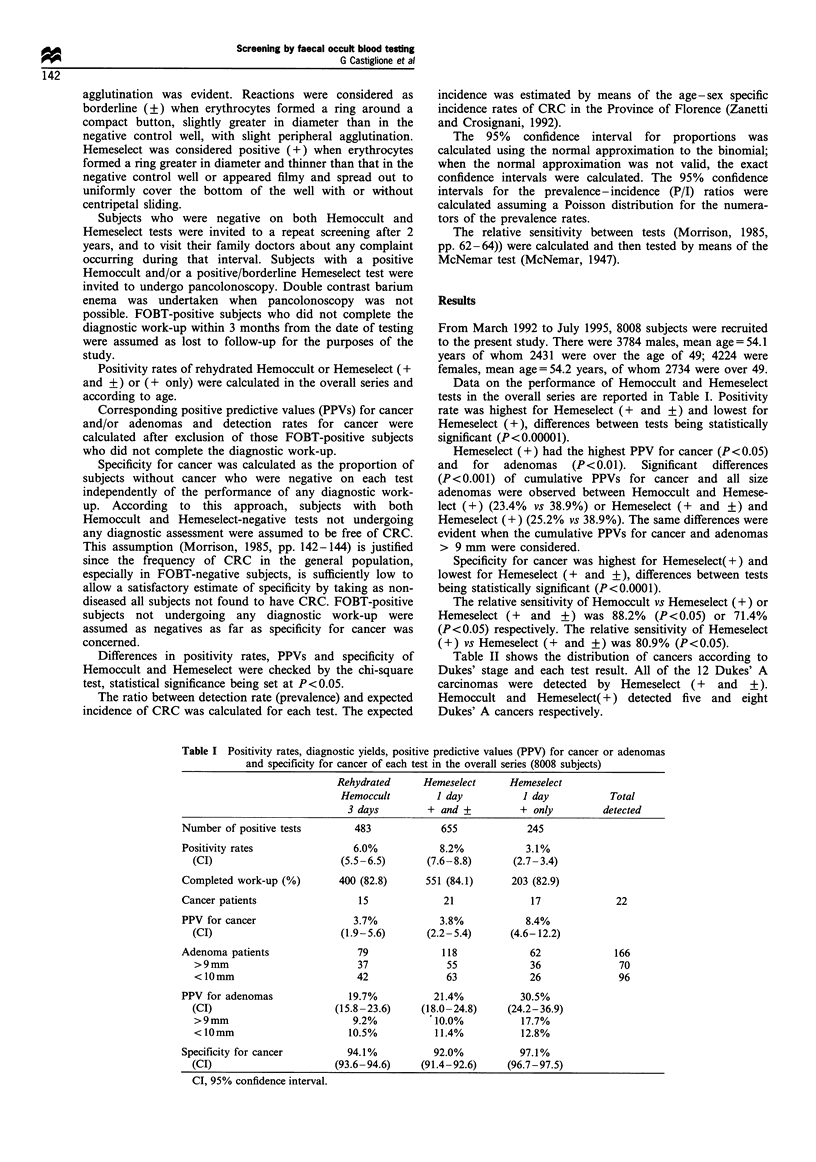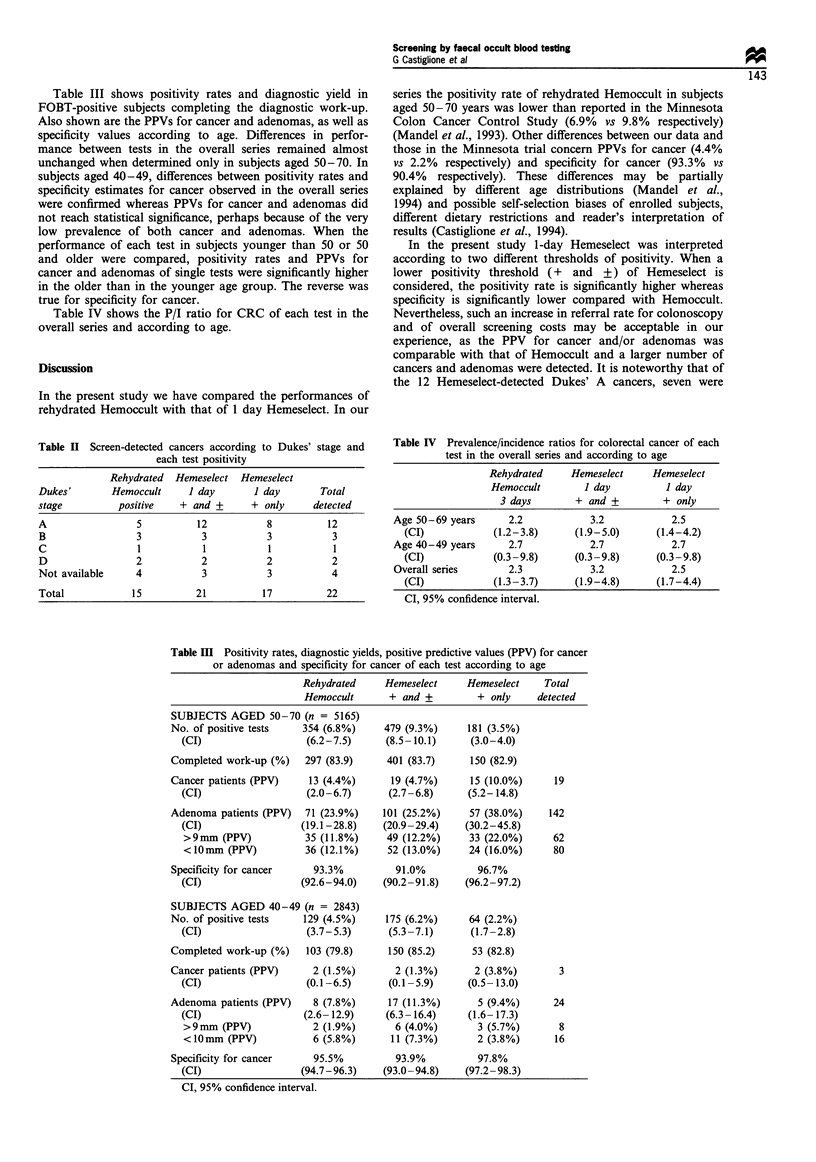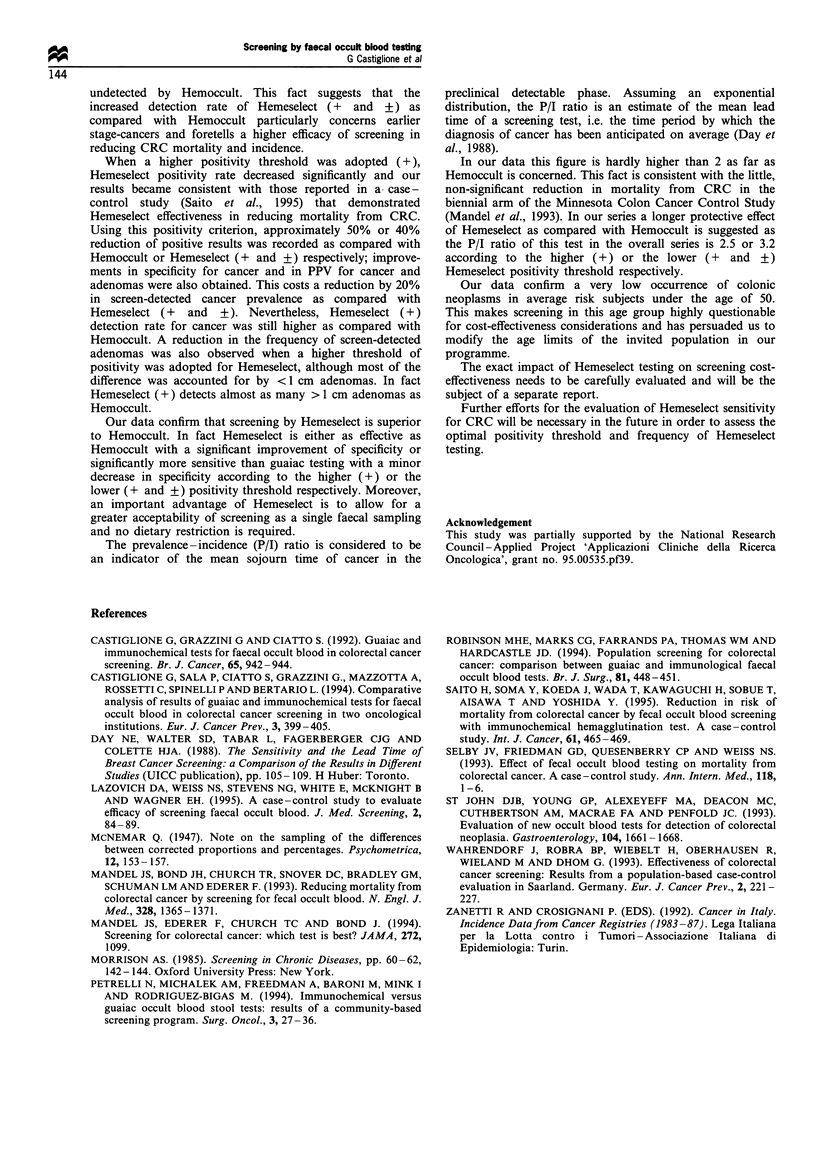# Immunochemical vs guaiac faecal occult blood tests in a population-based screening programme for colorectal cancer.

**DOI:** 10.1038/bjc.1996.329

**Published:** 1996-07

**Authors:** G. Castiglione, M. Zappa, G. Grazzini, A. Mazzotta, M. Biagini, P. Salvadori, S. Ciatto

**Affiliations:** Centro per lo Studio e la Prevenzione Oncologica, Florence, Italy

## Abstract

Two faecal occult blood tests (FOBTs), Hemoccult II (guaiac based) and Hemeselect (immunochemical) were compared in a population screening for colorectal cancer on 24 282 subjects aged 40-70. Hemeselect was interpreted according to a lower (+ and +/-) and a higher (+) positivity threshold. A total of 8008 compliers were enrolled in the study. Positivity rates: Hemoccult = 6.0%, Hemeselect (+ and +/) = 8.2%, Hemeselect (+) = 3.1%. Among FOBT-positive subject complying with the diagnostic work-up, 22 had colorectal cancer (17 Hemeselect-positive (+), four Hemeselect-borderline (+/-), 15 Hemoccult-positive) and 166 subjects had adenomas (62 Hemeselect(+), 56 Hemeselect-borderline (+/-), 79 Hemoccult-positive) were detected. The positive predictive values (PPVs) for cancer were as follows: Hemoccult = 3.7%, Hemeselect (+ and +/-) = 3.8%, Hemeselect (+) = 8.4%. The PPVs for adenoma(s) were: Hemoccult = 19.7%, Hemeselect (+ and +/-) = 21.4%, Hemeselect (+) = 30.5%. The specificity for cancer was: Hemoccult = 94.1%, Hemeselect (+ +/-) = 92%, Hemeselect (+) = 97.1%. Ratios between detection rates of each test and expected incidence of colorectal cancer suggest that Hemoccult anticipates cancer diagnosis by approximately 2 years on average whereas the mean diagnostic anticipation of Hemeselect ranges between 2.5 and 3.2 years. Hemeselect is superior to Hemoccult as it is at least as effective but more efficient and acceptable than guaiac testing. Further evaluation of Hemeselect cost-effectiveness and sensitivity is needed in order to assess the optimal threshold of positivity and screening frequency.


British Journal of Cancer (1996) 74, 141-144
© 1996 Stockton Press All rights reserved 0007-0920/96 $12.00

### Immunochemical *vs* guaiac faecal occult blood tests in a population-based screening programme for colorectal cancer

G Castiglione[1], M Zappa[1], G Grazzini[1], A Mazzotta[1], M Biagini[2], P Salvadori[3] and S Ciatto[1]

[1]*Centro per lo Studio e la Prevenzione Oncologica, Viale A Volta 171, I-50131, Italy;* [2]*Endoscopy Unit, Ospedale Santa Verdiana, Viale dei Mille 1, I-50051 Castelfiorentino, Italy;* [3]*Unità Sanitaria Locale 11, Piazza XXIV Luglio 1, I-50053 Empoli, Italy.*

**Summary** Two faecal occult blood tests (FOBTs), Hemoccult II (guaiac based) and Hemeselect (immunochemical) were compared in a population screening for colorectal cancer on 24 282 subjects aged 40-70. Hemeselect was interpreted according to a lower (+ and ±) and a higher (+) positivity threshold. A total of 8008 compliers were enrolled in the study. Positivity rates: Hemoccult=6.0%, Hemeselect (+ and ±)=8.2%, Hemeselect (+)=3.1%. Among FOBT-positive subjects complying with the diagnostic work-up, 22 had colorectal cancer (17 Hemeselect-positive (+), four Hemeselect-borderline (±), 15 Hemoccult-positive) and 166 subjects had adenomas (62 Hemeselect(+), 56 Hemeselect-borderline (±), 79 Hemoccult-positive) were detected. The positive predictive values (PPVs) for cancer were as follows: Hemoccult=3.7%, Hemeselect (+ and ±)=3.8%, Hemeselect (+)=8.4%. The PPVs for adenoma(s) were: Hemoccult=19.7%, Hemeselect (+ and ±)=21.4%, Hemeselect (+)=30.5%. The specificity for cancer was: Hemoccult=94.1%, Hemeselect (+ and ±)=92%, Hemeselect (+)=97.1%. Ratios between detection rates of each test and expected incidence of colorectal cancer suggest that Hemoccult anticipates cancer diagnosis by approximately 2 years on average whereas the mean diagnostic anticipation of Hemeselect ranges between 2.5 and 3.2 years. Hemeselect is superior to Hemoccult as it is at least as effective but more efficient and acceptable than guaiac testing. Further evaluation of Hemeselect cost-effectiveness and sensitivity is needed in order to assess the optimal threshold of positivity and screening frequency.

**Keywords:** colorectal neoplasms-prevention and control; colorectal neoplasms-diagnosis; occult blood; guaiac test; immunochemical test

In recent years several randomised (Mandel *et al.*, 1993) or case-control studies (Selby *et al.*, 1993; Wahrenrdorf *et al.*, 1993; Lazovich *et al.*, 1995) have reported guaiac-based faecal occult blood testing (Hemoccult II, SmithKline Diagnostics, San Jose, CA, USA) to reduce mortality from colorectal cancer (CRC). The Minnesota Colon Cancer Control Study (Mandel *et al.*, 1993) has demonstrated that annual screening by rehydrated Hemoccult II is able to achieve a 33% reduction of mortality from CRC. Thus rehydrated Hemoccult may be taken to be the standard test for comparison of any other new faecal occult blood test (FOBT) in CRC screening. In the above-mentioned study rehydrated Hemoccult shows a high sensitivity for CRC (92.2%) but the corresponding specificity value is disappointingly low (90.4%), causing a high referral rate to colonoscopy (9.8%) and a relevant increase in screening costs. Another test, Hemeselect (SmithKline Diagnostics, San Jose, CA, USA), based on reverse-passive haemagglutination, has been (found to show) increased sensitivity as compared with Hemoccult (Castiglione *et al.*, 1992; St John *et al.*, 1993; Petrelli *et al.*, 1994; Castiglione *et al.*, 1994; Robinson *et al.*, 1994) and also evidence of effectiveness in reducing mortality from CRC (Saito *et al.*, 1995). In our preliminary experiment (Castiglione *et al.*, 1992; 1994) in self-referring subjects 1day Hemeselect testing showed a higher detection rate for cancer and adenomas as compared with 3 day rehydrated Hemoccult whereas specificity did not significantly differ. These results suggest that 1 day Hemeselect may be more accurate than rehydrated Hemoccult. Moreover, Hemeselect requires no dietary restriction, a condition which is likely to increase the compliance with screening.

The aim of the present study is to compare the accuracy of 1 day Hemeselect and 3 day rehydrated Hemoccult in a population-based screening programme in the Province of Florence.

### Methods

A population-based screening programme for CRC by Hemoccult has been in progress since 1982 in 28 municipalities in the province of Florence. All subjects aged 40-70 living in the screening area have been invited every other year to undergo the screening protocol, run by the Centro per lo Studio e la Prevenzione Oncologica of Florence.

From March 1992, screening was initiated in four newly involved municipalities (14 682 inhabitants aged 40-70) with a new protocol based on two FOBTs. Attendance rates ranged between 38% and 48% (mean=44%). In 1994 the new protocol was introduced in two municipalities previously screened by Hemoccult alone (9 600 subjects aged 40-70). Attendance rates in these two municipalities were 37% and 38% (mean=37.5%).

In the present study only the first attendance with the new protocol was considered for each subject, provided both tests had been completed. Subjects previously screened by Hemoccult alone were included in our study provided at least 3 years had elapsed since the most recent screening.

Attenders were asked to collect faeces samples using Hemoccult kits on three consecutive bowel movement and Hemeselect kits on the first bowel movement only. Compliers were invited not to eat red meat 2 days before and during faeces samples collection. Returned specimens were developed in our laboratory usually within 1 week from faeces samples collection. Hemoccult was developed after rehydration and was considered positive when a blue colour appeared at least in one slide after application of one drop of developer. Hemeselect was interpreted at 1/8 dilution according to two positivity thresholds: positive (+) or borderline (±). After erythrocytes coated with anti-human haemoglobin antibodies were added to the diluted extract of faecal specimens, Hemeselect reactions were considered negative when no

Correspondence: G Castiglione, Centro per lo Studio e la Prevenzione Oncologica, Viale A Volta 171, I-50131, Florence, Italy
Received 1 December 1995; revised 25 January 1996; accepted 25 January 1996

agglutination was evident. Reactions were considered as borderline (±) when erythrocytes formed a ring around a compact button, slightly greater in diameter than in the negative control well, with slight peripheral agglutination. Hemeselect was considered positive (+) when erythrocytes formed a ring greater in diameter and thinner than that in the negative control well or appeared filmy and spread out to uniformly cover the bottom of the well with or without centripetal sliding.

Subjects who were negative on both Hemoccult and Hemeselect tests were invited to a repeat screening after 2 years, and to visit their family doctors about any complaint occurring during that interval. Subjects with a positive Hemoccult and/or a positive/borderline Hemeselect test were invited to undergo pancolonoscopy. Double contrast barium enema was undertaken when pancolonoscopy was not possible. FOBT-positive subjects who did not complete the diagnostic work-up within 3 months from the date of testing were assumed as lost to follow-up for the purposes of the study.

Positivity rates of rehydrated Hemoccult or Hemeselect (+ and ±) or (+ only) were calculated in the overall series and according to age.

Corresponding positive predictive values (PPVs) for cancer and/or adenomas and detection rates for cancer were calculated after exclusion of those FOBT-positive subjects who did not complete the diagnostic work-up.

Specificity for cancer was calculated as the proportion of subjects without cancer who were negative on each test independently of the performance of any diagnostic work-up. According to this approach, subjects with both Hemoccult and Hemeselect-negative tests not undergoing any diagnostic assessment were assumed to be free of CRC. This assumption (Morrison, 1985, pp. 142–144) is justified since the frequency of CRC in the general population, especially in FOBT-negative subjects, is sufficiently low to allow a satisfactory estimate of specificity by taking as non-diseased all subjects not found to have CRC. FOBT-positive subjects not undergoing any diagnostic work-up were assumed as negatives as far as specificity for cancer was concerned.

Differences in positivity rates, PPVs and specificity of Hemoccult and Hemeselect were checked by the chi-square test, statistical significance being set at $P<0.05$.

The ratio between detection rate (prevalence) and expected incidence of CRC was calculated for each test. The expected incidence was estimated by means of the age–sex specific incidence rates of CRC in the Province of Florence (Zanetti and Crosignani, 1992).

The 95% confidence interval for proportions was calculated using the normal approximation to the binomial; when the normal approximation was not valid, the exact confidence intervals were calculated. The 95% confidence intervals for the prevalence–incidence (P/I) ratios were calculated assuming a Poisson distribution for the numerators of the prevalence rates.

The relative sensitivity between tests (Morrison, 1985, pp. 62–64)) were calculated and then tested by means of the McNemar test (McNemar, 1947).

### Results

From March 1992 to July 1995, 8008 subjects were recruited to the present study. There were 3784 males, mean age = 54.1 years of whom 2431 were over the age of 49; 4224 were females, mean age = 54.2 years, of whom 2734 were over 49.

Data on the performance of Hemoccult and Hemeselect tests in the overall series are reported in Table I. Positivity rate was highest for Hemeselect (+ and ±) and lowest for Hemeselect (+), differences between tests being statistically significant ($P<0.00001$).

Hemeselect (+) had the highest PPV for cancer ($P<0.05$) and for adenomas ($P<0.01$). Significant differences ($P<0.001$) of cumulative PPVs for cancer and all size adenomas were observed between Hemoccult and Hemeselect (+) (23.4% *vs* 38.9%) or Hemeselect (+ and ±) and Hemeselect (+) (25.2% *vs* 38.9%). The same differences were evident when the cumulative PPVs for cancer and adenomas > 9 mm were considered.

Specificity for cancer was highest for Hemeselect(+) and lowest for Hemeselect (+ and ±), differences between tests being statistically significant ($P<0.0001$).

The relative sensitivity of Hemoccult *vs* Hemeselect (+) or Hemeselect (+ and ±) was 88.2% ($P<0.05$) or 71.4% ($P<0.05$) respectively. The relative sensitivity of Hemeselect (+) *vs* Hemeselect (+ and ±) was 80.9% ($P<0.05$).

Table II shows the distribution of cancers according to Dukes' stage and each test result. All of the 12 Dukes' A carcinomas were detected by Hemeselect (+ and ±). Hemoccult and Hemeselect(+) detected five and eight Dukes' A cancers respectively.

**Table I** Positivity rates, diagnostic yields, positive predictive values (PPV) for cancer or adenomas and specificity for cancer of each test in the overall series (8008 subjects)

| | *Rehydrated Hemoccult 3 days* | *Hemeselect 1 day + and ±* | *Hemeselect 1 day + only* | *Total detected* |
|---|---|---|---|---|
| Number of positive tests | 483 | 655 | 245 | |
| Positivity rates | 6.0% | 8.2% | 3.1% | |
| (CI) | (5.5–6.5) | (7.6–8.8) | (2.7–3.4) | |
| Completed work-up (%) | 400 (82.8) | 551 (84.1) | 203 (82.9) | |
| Cancer patients | 15 | 21 | 17 | 22 |
| PPV for cancer | 3.7% | 3.8% | 8.4% | |
| (CI) | (1.9–5.6) | (2.2–5.4) | (4.6–12.2) | |
| Adenoma patients | 79 | 118 | 62 | 166 |
| >9 mm | 37 | 55 | 36 | 70 |
| <10 mm | 42 | 63 | 26 | 96 |
| PPV for adenomas | 19.7% | 21.4% | 30.5% | |
| (CI) | (15.8–23.6) | (18.0–24.8) | (24.2–36.9) | |
| >9 mm | 9.2% | 10.0% | 17.7% | |
| <10 mm | 10.5% | 11.4% | 12.8% | |
| Specificity for cancer | 94.1% | 92.0% | 97.1% | |
| (CI) | (93.6–94.6) | (91.4–92.6) | (96.7–97.5) | |

CI, 95% confidence interval.

Table III shows positivity rates and diagnostic yield in FOBT-positive subjects completing the diagnostic work-up. Also shown are the PPVs for cancer and adenomas, as well as specificity values according to age. Differences in performance between tests in the overall series remained almost unchanged when determined only in subjects aged 50–70. In subjects aged 40–49, differences between positivity rates and specificity estimates for cancer observed in the overall series were confirmed whereas PPVs for cancer and adenomas did not reach statistical significance, perhaps because of the very low prevalence of both cancer and adenomas. When the performance of each test in subjects younger than 50 or 50 and older were compared, positivity rates and PPVs for cancer and adenomas of single tests were significantly higher in the older than in the younger age group. The reverse was true for specificity for cancer.

Table IV shows the P/I ratio for CRC of each test in the overall series and according to age.

### Discussion

In the present study we have compared the performances of rehydrated Hemoccult with that of 1 day Hemeselect. In our series the positivity rate of rehydrated Hemoccult in subjects aged 50–70 years was lower than reported in the Minnesota Colon Cancer Control Study (6.9% *vs* 9.8% respectively) (Mandel *et al.*, 1993). Other differences between our data and those in the Minnesota trial concern PPVs for cancer (4.4% *vs* 2.2% respectively) and specificity for cancer (93.3% *vs* 90.4% respectively). These differences may be partially explained by different age distributions (Mandel *et al.*, 1994) and possible self-selection biases of enrolled subjects, different dietary restrictions and reader's interpretation of results (Castiglione *et al.*, 1994).

In the present study 1-day Hemeselect was interpreted according to two different thresholds of positivity. When a lower positivity threshold (+ and ±) of Hemeselect is considered, the positivity rate is significantly higher whereas specificity is significantly lower compared with Hemoccult. Nevertheless, such an increase in referral rate for colonoscopy and of overall screening costs may be acceptable in our experience, as the PPV for cancer and/or adenomas was comparable with that of Hemoccult and a larger number of cancers and adenomas were detected. It is noteworthy that of the 12 Hemeselect-detected Dukes' A cancers, seven were

**Table II** Screen-detected cancers according to Dukes' stage and each test positivity

| *Dukes' stage* | *Rehydrated Hemoccult positive* | *Hemeselect 1 day + and ±* | *Hemeselect 1 day + only* | *Total detected* |
|---|---|---|---|---|
| A | 5 | 12 | 8 | 12 |
| B | 3 | 3 | 3 | 3 |
| C | 1 | 1 | 1 | 1 |
| D | 2 | 2 | 2 | 2 |
| Not available | 4 | 3 | 3 | 4 |
| Total | 15 | 21 | 17 | 22 |

**Table IV** Prevalence/incidence ratios for colorectal cancer of each test in the overall series and according to age

| | *Rehydrated Hemoccult 3 days* | *Hemeselect 1 day + and ±* | *Hemeselect 1 day + only* |
|---|---|---|---|
| Age 50–69 years | 2.2 | 3.2 | 2.5 |
| (CI) | (1.2–3.8) | (1.9–5.0) | (1.4–4.2) |
| Age 40–49 years | 2.7 | 2.7 | 2.7 |
| (CI) | (0.3–9.8) | (0.3–9.8) | (0.3–9.8) |
| Overall series | 2.3 | 3.2 | 2.5 |
| (CI) | (1.3–3.7) | (1.9–4.8) | (1.7–4.4) |

CI, 95% confidence interval.

**Table III** Positivity rates, diagnostic yields, positive predictive values (PPV) for cancer or adenomas and specificity for cancer of each test according to age

| | *Rehydrated Hemoccult* | *Hemeselect + and ±* | *Hemeselect + only* | *Total detected* |
|---|---|---|---|---|
| SUBJECTS AGED 50–70 (*n* = 5165) | | | | |
| No. of positive tests | 354 (6.8%) | 479 (9.3%) | 181 (3.5%) | |
| (CI) | (6.2–7.5) | (8.5–10.1) | (3.0–4.0) | |
| Completed work-up (%) | 297 (83.9) | 401 (83.7) | 150 (82.9) | |
| Cancer patients (PPV) | 13 (4.4%) | 19 (4.7%) | 15 (10.0%) | 19 |
| (CI) | (2.0–6.7) | (2.7–6.8) | (5.2–14.8) | |
| Adenoma patients (PPV) | 71 (23.9%) | 101 (25.2%) | 57 (38.0%) | 142 |
| (CI) | (19.1–28.8) | (20.9–29.4) | (30.2–45.8) | |
| >9 mm (PPV) | 35 (11.8%) | 49 (12.2%) | 33 (22.0%) | 62 |
| <10 mm (PPV) | 36 (12.1%) | 52 (13.0%) | 24 (16.0%) | 80 |
| Specificity for cancer | 93.3% | 91.0% | 96.7% | |
| (CI) | (92.6–94.0) | (90.2–91.8) | (96.2–97.2) | |
| SUBJECTS AGED 40–49 (*n* = 2843) | | | | |
| No. of positive tests | 129 (4.5%) | 175 (6.2%) | 64 (2.2%) | |
| (CI) | (3.7–5.3) | (5.3–7.1) | (1.7–2.8) | |
| Completed work-up (%) | 103 (79.8) | 150 (85.2) | 53 (82.8) | |
| Cancer patients (PPV) | 2 (1.5%) | 2 (1.3%) | 2 (3.8%) | 3 |
| (CI) | (0.1–6.5) | (0.1–5.9) | (0.5–13.0) | |
| Adenoma patients (PPV) | 8 (7.8%) | 17 (11.3%) | 5 (9.4%) | 24 |
| (CI) | (2.6–12.9) | (6.3–16.4) | (1.6–17.3) | |
| >9 mm (PPV) | 2 (1.9%) | 6 (4.0%) | 3 (5.7%) | 8 |
| <10 mm (PPV) | 6 (5.8%) | 11 (7.3%) | 2 (3.8%) | 16 |
| Specificity for cancer | 95.5% | 93.9% | 97.8% | |
| (CI) | (94.7–96.3) | (93.0–94.8) | (97.2–98.3) | |

CI, 95% confidence interval.

undetected by Hemoccult. This fact suggests that the increased detection rate of Hemeselect (+ and ±) as compared with Hemoccult particularly concerns earlier stage-cancers and foretells a higher efficacy of screening in reducing CRC mortality and incidence.

When a higher positivity threshold was adopted (+), Hemeselect positivity rate decreased significantly and our results became consistent with those reported in a case–control study (Saito *et al.*, 1995) that demonstrated Hemeselect effectiveness in reducing mortality from CRC. Using this positivity criterion, approximately 50% or 40% reduction of positive results was recorded as compared with Hemoccult or Hemeselect (+ and ±) respectively; improvements in specificity for cancer and in PPV for cancer and adenomas were also obtained. This costs a reduction by 20% in screen-detected cancer prevalence as compared with Hemeselect (+ and ±). Nevertheless, Hemeselect (+) detection rate for cancer was still higher as compared with Hemoccult. A reduction in the frequency of screen-detected adenomas was also observed when a higher threshold of positivity was adopted for Hemeselect, although most of the difference was accounted for by <1 cm adenomas. In fact Hemeselect (+) detects almost as many >1 cm adenomas as Hemoccult.

Our data confirm that screening by Hemeselect is superior to Hemoccult. In fact Hemeselect is either as effective as Hemoccult with a significant improvement of specificity or significantly more sensitive than guaiac testing with a minor decrease in specificity according to the higher (+) or the lower (+ and ±) positivity threshold respectively. Moreover, an important advantage of Hemeselect is to allow for a greater acceptability of screening as a single faecal sampling and no dietary restriction is required.

The prevalence–incidence (P/I) ratio is considered to be an indicator of the mean sojourn time of cancer in the preclinical detectable phase. Assuming an exponential distribution, the P/I ratio is an estimate of the mean lead time of a screening test, i.e. the time period by which the diagnosis of cancer has been anticipated on average (Day *et al.*, 1988).

In our data this figure is hardly higher than 2 as far as Hemoccult is concerned. This fact is consistent with the little, non-significant reduction in mortality from CRC in the biennial arm of the Minnesota Colon Cancer Control Study (Mandel *et al.*, 1993). In our series a longer protective effect of Hemeselect as compared with Hemoccult is suggested as the P/I ratio of this test in the overall series is 2.5 or 3.2 according to the higher (+) or the lower (+ and ±) Hemeselect positivity threshold respectively.

Our data confirm a very low occurrence of colonic neoplasms in average risk subjects under the age of 50. This makes screening in this age group highly questionable for cost-effectiveness considerations and has persuaded us to modify the age limits of the invited population in our programme.

The exact impact of Hemeselect testing on screening cost-effectiveness needs to be carefully evaluated and will be the subject of a separate report.

Further efforts for the evaluation of Hemeselect sensitivity for CRC will be necessary in the future in order to assess the optimal positivity threshold and frequency of Hemeselect testing.

**Acknowledgement**
This study was partially supported by the National Research Council–Applied Project 'Applicazioni Cliniche della Ricerca Oncologica', grant no. 95.00535.pf39.

### References

CASTIGLIONE G, GRAZZINI G AND CIATTO S. (1992). Guaiac and immunochemical tests for faecal occult blood in colorectal cancer screening. *Br. J. Cancer*, **65**, 942–944.

CASTIGLIONE G, SALA P, CIATTO S, GRAZZINI G., MAZZOTTA A, ROSSETTI C, SPINELLI P AND BERTARIO L. (1994). Comparative analysis of results of guaiac and immunochemical tests for faecal occult blood in colorectal cancer screening in two oncological institutions. *Eur. J. Cancer Prev.*, **3**, 399–405.

DAY NE, WALTER SD, TABAR L, FAGERBERGER CJG AND COLETTE HJA. (1988). *The Sensitivity and the Lead Time of Breast Cancer Screening: a Comparison of the Results in Different Studies* (UICC publication), pp. 105–109. H Huber: Toronto.

LAZOVICH DA, WEISS NS, STEVENS NG, WHITE E, McKNIGHT B AND WAGNER EH. (1995). A case–control study to evaluate efficacy of screening faecal occult blood. *J. Med. Screening*, **2**, 84–89.

McNEMAR Q. (1947). Note on the sampling of the differences between corrected proportions and percentages. *Psychometrica*, **12**, 153–157.

MANDEL JS, BOND JH, CHURCH TR, SNOVER DC, BRADLEY GM, SCHUMAN LM AND EDERER F. (1993). Reducing mortality from colorectal cancer by screening for fecal occult blood. *N. Engl. J. Med.*, **328**, 1365–1371.

MANDEL JS, EDERER F, CHURCH TC AND BOND J. (1994). Screening for colorectal cancer: which test is best? *JAMA*, **272**, 1099.

MORRISON AS. (1985). *Screening in Chronic Diseases*, pp. 60–62, 142–144. Oxford University Press: New York.

PETRELLI N, MICHALEK AM, FREEDMAN A, BARONI M, MINK I AND RODRIGUEZ-BIGAS M. (1994). Immunochemical versus guaiac occult blood stool tests: results of a community-based screening program. *Surg. Oncol.*, **3**, 27–36.

ROBINSON MHE, MARKS CG, FARRANDS PA, THOMAS WM AND HARDCASTLE JD. (1994). Population screening for colorectal cancer: comparison between guaiac and immunological faecal occult blood tests. *Br. J. Surg.*, **81**, 448–451.

SAITO H, SOMA Y, KOEDA J, WADA T, KAWAGUCHI H, SOBUE T, AISAWA T AND YOSHIDA Y. (1995). Reduction in risk of mortality from colorectal cancer by fecal occult blood screening with immunochemical hemagglutination test. A case–control study. *Int. J. Cancer*, **61**, 465–469.

SELBY JV, FRIEDMAN GD, QUESENBERRY CP AND WEISS NS. (1993). Effect of fecal occult blood testing on mortality from colorectal cancer. A case–control study. *Ann. Intern. Med.*, **118**, 1–6.

ST JOHN DJB, YOUNG GP, ALEXEYEFF MA, DEACON MC, CUTHBERTSON AM, MACRAE FA AND PENFOLD JC. (1993). Evaluation of new occult blood tests for detection of colorectal neoplasia. *Gastroenterology*, **104**, 1661–1668.

WAHRENDORF J, ROBRA BP, WIEBELT H, OBERHAUSEN R, WIELAND M AND DHOM G. (1993). Effectiveness of colorectal cancer screening: Results from a population-based case-control evaluation in Saarland. Germany. *Eur. J. Cancer Prev.*, **2**, 221–227.

ZANETTI R AND CROSIGNANI P. (EDS). (1992). *Cancer in Italy. Incidence Data from Cancer Registries (1983–87)*. Lega Italiana per la Lotta contro i Tumori–Associazione Italiana di Epidemiologia: Turin.